# Preparation of Polyurea Microcapsules by Interfacial Polymerization of Isocyanate and Chitosan Oligosaccharide

**DOI:** 10.3390/ma14133753

**Published:** 2021-07-05

**Authors:** Fuqiang Yu, Ying Wang, Yan Zhao, Jingyu Chou, Xiaowu Li

**Affiliations:** 1 Department of Materials Physics and Chemistry, School of Materials Science and Engineering, and Key Laboratory for Anisotropy and Texture of Materials, Ministry of Education, Northeastern University, Shenyang 110819, china; yufuqiang2003@163.com; 2 State Key Laboratory for the Discovery and Development of Novel Pesticides, Shenyang Sinochem Agrochemicals R&D Co., Ltd., Shenyang 110021, China; wangying09@sinochem.com (Y.W.); choujingyu@sinochem.com (J.C.); 3 Jihua Laboratory, Nanhai, Foshan 528200, China

**Keywords:** polyurea, microcapsule, chitosan oligosaccharide, pesticides, slow release

## Abstract

(2-((1-(4-chlorophenyl)-1*H*-pyrazol-3-yl)oxy)-*N*-(3,4-dichlorophenyl)-propanamide) is a new oil-soluble compound with good fungicidal activity against *Rhizoctonia solani*. Chitosan oligosaccharide (COS) is the depolymerization product of chitosan and can be developed into biological pesticides, growth regulators, and fertilizers due to its various bioactivities. COS is an oligomer of β- (1 → 4)-linked d –glucosamine and can be taken as a polyamine. In this study, microcapsules were prepared by interfacial polymerization of oil-soluble methylene diphenyl diisocyanate and water-soluble COS. The effects of several key preparation parameters, e.g., emulsifier dosage, agitation rate during emulsification, and core/shell ratio, on properties of the microcapsules such as the encapsulation efficiency, particle size, and size distribution were investigated. The microcapsules were characterized by infrared spectroscopy, thermogravimetric analysis, and scanning electron microscopy, etc., and the encapsulation efficiency and release behaviors were investigated. The results show that the microcapsules have a smooth surface and 93.3% of encapsulation efficiency. The microcapsules showed slow-release behavior following a first-order kinetic equation, and the accumulative release rates of the microcapsules with core/shell mass ratios of 8.0/4.0, 8.0/5.0, and 8.0/6.0, were 95.5%, 91.4%, and 90.1%, respectively, on day 30. Due to many high biological activities, biodegradability, and the pure nature of COS, microcapsules formed from COS are promising for applications in controlled release of pesticides, growth regulators, and fertilizer.

## 1. Introduction

As one of the major diseases, rice sheath blight is caused by *Rhizoctonia solani* Kühn and occurs every year in the word, resulting in a large reduction of rice yield [1]. As it can occur in the whole growth stage of rice, it is necessary to use carriers to release the fungicide slowly in order to cope with the lasting effect of rice sheath blight. One of the most important forms of slow-release carriers is microcapsules, which have core—shell structure cores where active ingredients are coated with natural or synthetic polymers, i.e., shell materials, by physical, chemical, or physicochemical methods [2] and have a range of desirable properties [3,4,5,6]. (i) The core active ingredient can be released at a designed rate, which enables improved deinsectization, weed killing, and fungi inhibition. (ii) Decomposition of pesticides caused by environmental factors such as light, air, and microorganisms is reduced. (iii) The pesticide efficacy is prolonged, which saves labor and reduces administration times. (iv) Direct contact between the pesticide and the human body is decreased, unpleasant odors are masked or decreased, and drifting and poisoning of non-target organisms are decreased. The encapsulation of pesticides such as cypermethrin, phoxim, chlorpyrifos, abamectin, pyraclostrobin, and clomazone have been studied for use in commercial pesticide microcapsule release agents [7,8,9].

Many materials have been studied for use as shell materials in pesticide microcapsules. Polyurea is generally used because of properties such as high stability and mechanical strength, low cost, and good release characteristics [8,10,11]. Polyurea, which contains strongly polar urea groups (–NHCONH–), can be synthesized from polyisocyanates and polyamines. The most widely used polyisocyanates are polyaryl polymethylene isocyanate, isophorone diisocyanate (IPDI), hexamethylene diisocyanate, methylene diphenyl diisocyanate (MDI), dicyclohexylmethane-4,4′-diisocyanate, and toluene diisocyanate (TDI). Commonly used polyamines are ethylenediamine, hexanediamine, triethylenetetramine, and diethylenetriamine [12,13,14,15,16,17,18,19]. For example, Jiang et al. [13] prepared polyurea microcapsules containing *n*-octadecane by using IPDI and diethylenetriamine as the monomers. Zhang et al. [16] fabricated chlorpyrifos microcapsules with a polyurea shell synthesized from TDI and ethylenediamine.

Polyamines have several disadvantages, however, as the water-soluble reactants in microcapsule preparation, e.g., toxicity, volatility, and flammability, can damage the environment. Greenness, environmental protection, and sustainable development are now of great importance, and the development of new, environmentally friendly reagents is urgently needed.

**Figure 1:**
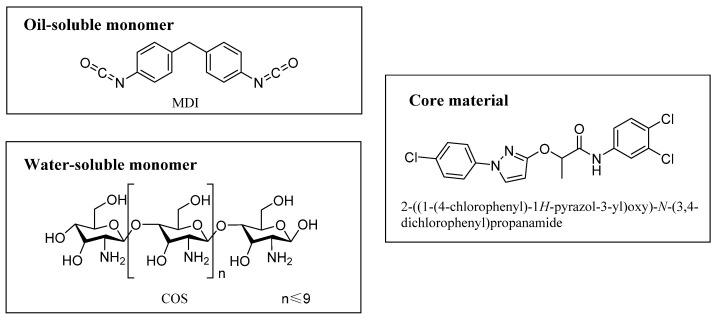
Molecular structures of monomers and core material used in this study.

Chitosan is a partial deacetylation product of chitin and consists of randomly distributed β-(1 → 4)-linked D-glucosamine units (deacetylated unit) and *N*-acetyl- D-glucosamine (acetylated unit). Chitin is widely found in the exoskeletons of arthropods and insects, and in the cell walls of fungi. Chitosan oligosaccharide (COS) is the product of enzymatic or chemical depolymerization of chitosan; its structure is shown in Figure 1. Some of the properties of COS differ from those of chitosan, e.g., it has good aqueous solubility. It has anti-inflammatory, anticancer, antioxidant, and antifungal properties and has been widely used in medical applications, animal feed, fertilizers, and pesticides [20,21,22,23,24,25,26,27,28,29,30,31]. In the field of crop protection, COS has been shown to change the soil flora, promote the growth of beneficial microorganisms, and induce disease resistance of plants; it can be used in biological pesticides, growth regulators, and fertilizers [20,32,33,34,35]. Li et al. [36] also reported that treatment of seeds with a COS solution increased the yields of grain crops, vegetables, and fruits by 10–30%. Benzophenonetetracarboxylic dianhydride-grafted COS has been studied as the membrane material for preparing UV-resistant polyurea microcapsules for encapsulating Avermectin [37].

2-((1-(4-chlorophenyl)-1*H*-pyrazol-3-yl)oxy)-*N*-(3,4-dichlorophenyl)propenamide, as shown in Figure 1, is a new compound reported by our group with good fungicidal activity against *Rhizoctonia solani* [38]. The objective of this study was to prepare polyurea microcapsules by using COS as the water-soluble chain extender and 2-((1-(4-chlorophenyl)-1*H*-pyrazol-3-yl)oxy)-*N*-(3,4-dichlorophenyl)propanamide as the core materials. The pesticide microcapsules have the following characteristics: (i) slow, controlled-release behavior; (ii) environmental friendliness because of the odorlessness, biodegradability, and non-toxicity of COS; (iii) the ability to promote plant growth, due to the bioactivity of COS. Obtained microcapsules were characterized by infrared (IR) spectroscopy, thermogravimetric analysis (TGA), and scanning electron microscopy (SEM). The main factors that affect microcapsule preparation, e.g., emulsifier dosage, agitation rate, and core/shell ratio, were comprehensively investigated, and the release behavior of the core material was studied.

## 2. Materials, Instruments and Methods

### 2.1. Materials

The COS (molecular weight 1000 Da, 90% deacetylation degree) prepared by enzymolysis was kindly provided by the Institute of Process Engineering, Chinese Academy of Sciences. Other reagents were purchased through commercial vendors. Toluene (AR), methanol (AR) and *N*,*N*-dimethylformamide (DMF, AR) were purchased from Sinopharm Chemical Reagent Co., Ltd. Solvent oil S-150 (99%) was purchased from Zibo Shuoye Industry and Trade Co., Ltd. (Zibo, China) EO/PO block copolyether emulsion D800 (industrial pure) was purchased from Nanjing Golden Chemical Co., Ltd. (Nanjing, China). Polyvinyl alcohol emulsifier PVA-0588 (excellent grade) was purchased from Sinopec Sichuan Vinylon Works (Chongqing, China). Organic silicon defoamer DF-1540 (industrial pure) was purchased from Guangzhou Baiqian Chemical Co., Ltd (Guangzhou, China).

### 2.2. Instruments

The following instruments were used in the present study: JSM-7610F Plus (JEOL Ltd., Japan) and ZESIS SUPRA 35 (Carl Zeiss, Jena, Germany) scanning electron microscopes; Alpha II IR spectrometer equipped with an attenuated total reflection (ATR) attachment (Bruker, Bremen, Germany); STA 449 F3 Jupiter® thermal analyzer (NETZSCH-Gerätebau GmbH, Selb, Germany); LabTech ultraviolet (UV) spectrophotometer (LabTech Instrument Co., Ltd., Beijing, China) equipped with a 10 mm optical path length quartz cell; BT-2003 laser particle size distribution analyzer equipped with a BT-801 automatic cycle injection system and BT-1600 image granularity analysis system (Dandong Baite Instrument Co., Ltd., Dandong, China); YS100 optical microscope (Nikon Corporation, Tokyo, Japan); IKA T18 digital ULTRA-TURRAX high-speed shearing machine (IKA, Königswinter, Germany).

### 2.3. Microcapsule Preparation

**Figure 2:**
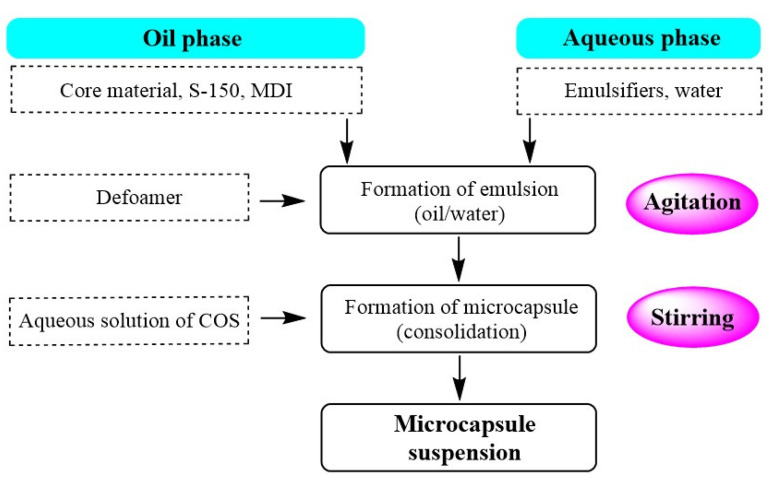
Preparation of microcapsules by interfacial polymerization.

Figure 2 shows a process for the preparation of microcapsules by interfacial polymerization. The core material, 2-((1-(4-chlorophenyl)-1*H*-pyrazol-3-yl)oxy)-*N*-(3,4-dichlorophenyl)-propanamide (4.00 g) and MDI were completely dissolved in S-150 (45.00 g) to generate a homogeneous organic phase. The aqueous phase was prepared by dissolving emulsifiers (a mixture of PVA-0588 and D800 at mass ratios of 1.0:1.0) in a certain amount of distilled water under stirring (the amount of water was adjusted according to the amounts of the other reagents to ensure that the mass of the whole system was 100 g). A few drops of the defoamer DF-1540 and the organic phase were added to the aqueous phase under vigorous high-speed stirring. An oil/water (O/W) emulsion formed within 5 min. The emulsion was immediately transferred to a three-necked flask, and a solution of COS in water (5.0 g) was added. The mixture was stirred for 20 min at room temperature. The temperature was then raised to 65 °C at a given heating rate, and the reaction was continued at this temperature for 6 h. After consolidation, a microcapsule suspension was obtained. The mass ratio of MDI to COS was 3 to 1. The experimental parameters, such as the emulsifier dosage, agitation rate, and core/shell ratio, were investigated by performing single-factor tests.

### 2.4. Characterization

#### 2.4.1. Measurement of Particle Size Distribution

The particle size distribution of the microcapsules was evaluated at room temperature by using a laser particle size analyzer. Each sample was measured 3 times, then the results were averaged. The relative span (RS) indicates the distribution width of the sample particle size. The smaller the RS is, the more concentrated the particle size dispersion of the microcapsules is. The RS can be calculated as [39]:

(1) $$\mathrm{RS}=\frac{D_{90}-D_{10}}{D_{50}}\;$$

where D_90_, D_10_ and D_50_ are diameters of 90%, 10% and 50% particle volume, respectively.

#### 2.4.2. Morphological Characterization

(1) Optical Microscopy (OM)

The dispersion states of the microcapsules were examined by OM. The obtained microcapsule dispersion was appropriately diluted with distilled water, spread on a microscope slide (2.5 cm × 7.5 cm), and covered with a cover glass. The samples were observed with a Nikon YS100 microscope.

(2) SEM

The surface morphology of the microcapsules was investigated by SEM. The sample was pre-treated as follows. The suspension was appropriately diluted with distilled water and then evenly coated on a thin layer of aluminum foil. After natural drying, the sample was adhered to the copper sample table with double-sided conductive tape and sprayed with platinum for 70 s before scanning. The surface morphology of the dried microcapsules was examined with a JSM-7610F Plus or ZESIS SUPRA 35 scanning electron microscope.

#### 2.4.3. IR Spectroscopy

IR spectra were recorded with a Bruker alpha II IR spectrometer equipped with an ATR accessory. The core material, COS, and MDI were each examined. The microcapsules were pre-treated as follows. The microcapsule dispersion was washed consecutively with methanol and water, centrifuged, and dried naturally. For each sample, 64 scans were performed between 400 and 4000 cm^−1^ at a resolution of 4 cm^−1^.

#### 2.4.4. Determination of Encapsulation Efficiency and Loading Capacity

For rapid analysis, a standard curve for the core material concentration and absorbance intensity was established. The procedure is listed in Supporting information, Figure S1 is the curve of core material absorbance versus wavelength, and Figure S2 is absorbance versus concentration calibration curve for core material at 284 nm. The relationship between the absorbance intensity (Abs) and mass concentration of the core material (C) is Abs = 0.04575C + 0.02038; for the standard curve, the correlation coefficient R^2^ = 0.99977.

(1) Encapsulation Efficiency Analysis

The encapsulation efficiency (EE) is defined as the percentage of core material incorporated into the microcapsules relative to the total amount of core material added (theoretical mass) during encapsulation, which reflects the degree of core material encapsulated by carrier (shell). The EE was determined by using a modified version of a previously published method [40]. Typically, a freshly prepared microcapsule suspension (2.5 g; d = 0.0001 g) and toluene (25 mL) were added to a flask; the flask was vibrated with an oscillating machine (150 r/min) for 20 min at room temperature, and the non-encapsulated core material was extracted. This extraction was repeated three times, and all the extracts were combined and filtered with a 0.45 μm organic membrane. After dilution with toluene to the required concentration, the clarified liquid was subjected to UV spectrometry. After determining the concentration of core material by using the standard curve and obtaining its mass, the EE was calculated as:

(2) $$\mathrm{EE}\left(\%\right)=\frac{\left(m_{0}-m_{1}\right)}{m_{0}}\times 100\%\;$$

where m_0_ is the theoretical mass of the core material and m_1_ is the mass of the non-encapsulated core material.

(2) Loading Capacity

The loading capacity (LC) is the percentage of the core material incorporated into a microcapsule relative to the total mass of the microcapsule (core and shell), which reflects the amount of core material contained in a unit mass of microcapsule; it was determined by using a previously reported method [41]. Freshly prepared microcapsule suspension was washed consecutively with methanol and water, centrifuged, and dried naturally. Two dried samples, each of mass 200 mg (d = 0.0001 g), were weighed out.

The first sample and toluene were placed in a 100 mL volumetric flask. This flask was vibrated with an oscillating machine (150 r/min) for 5 min at room temperature to extract the non-encapsulated core material.

The second sample was ground with a pestle. The sample was transferred to a 100 mL volumetric flask, dispersed in DMF (2 mL), and ultrasonicated for 10 min to break the microcapsules. Toluene was added to the mixture and the volumetric flask was vibrated with an oscillating machine (150 r/min) for 15 h to thoroughly extract the core material.

The toluene extracts were filtered with 0.45 μm organic membranes. Mass of core material in each sample was calculated from the obtained absorbance and the absorbance–concentration standard curve. LC was calculated as:

(3) $$\mathrm{LC}\left(\%\right)=\frac{\left(m_{2}-m_{1}\right)}{m_{0}}\times 100\%\;$$

where m_1_ is the mass of core material in the first sample, m_2_ is the mass of core material in the second sample, and m_0_ is the microcapsule mass.

#### 2.4.5. TGA

Thermal stability tests were performed with a NETZSCH STA 449 F3 Jupiter ® thermal analyzer (NETZSCH-Gerätebau GmbH, Selb, Germany). The core material was examined directly. The preparation of microcapsule powder referred to Section 2.4.3. Both experiments were performed with samples of mass about 5 mg at a heating rate of 3 °C/min in the range 30–500 °C with nitrogen as the purging and protective gas.

#### 2.4.6. 2-((1-(4-chlorophenyl)-1*H*-pyrazol-3-yl)oxy)-*N*-(3,4-dichlorophenyl)propanamide Release Tests

The release behavior was investigated with the method reported in the literature [8]. Small amounts of freshly prepared microcapsules were crushed and washed consecutively with methanol and water. Filtration and drying gave a dried microcapsule powder. A chromatography column (20 mm × 250 mm, with a sand plate) was filled from bottom to top with anhydrous sodium sulfate (3.0 g), 100-mesh silica gel (1.0 g), and the dried microcapsule powder (500 mg, d = 0.0001 g). From the second day, the filled column was eluted once every other day with 100 mL toluene. The absorbance of 2-((1-(4-chlorophenyl)-1*H*-pyrazol-3-yl)oxy)-*N*-(3,4-dichlorophenyl)propanamide was determined by UV spectroscopy, and its mass was calculated. The cumulative release rate Q was calculated as

(4) $$Q\left(\%\right)=\frac{\sum_{t=2}^{n}m_{t}}{m_{0}\times \mathrm{LC}}\times 100\%\;$$

where LC is the loading capacity, m_t_ is the mass of core material released on day t (t = 2, 4, 6, 8, 10… n, n = 30), and m_0_ is the mass of dried microcapsule powder used in the test.

## 3. Results and Discussion

Various factors that affect microcapsule preparation, e.g., the emulsifier dosage, heating rate during the consolidation process, agitation rate during emulsion preparation, and the core/shell ratio, were investigated in this study. Finally, TGA and release behavior of the microcapsule were studied.

### 3.1. Effects of Emulsifier Dosage on Encapsulation

The preparation of microcapsules by interfacial polymerization involves emulsification. A certain dosage of emulsifier is added during emulsion preparation to reduce the oil–water interfacial tension and produce a highly stable O/W emulsion [42]. In this study, the emulsifier used was a mixture of PVA-0588 and D800 in equal mass proportions. The emulsifier dosage greatly affects the particle size distribution. This was investigated by examining the microcapsule morphology.

**Figure 3:**
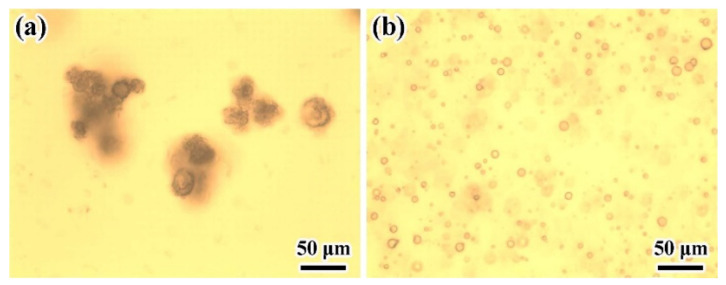
OM images of microcapsules prepared at different emulsifier dosages: (**a**) 0.5% and (**b**) 2.5%.

**Figure 4:**
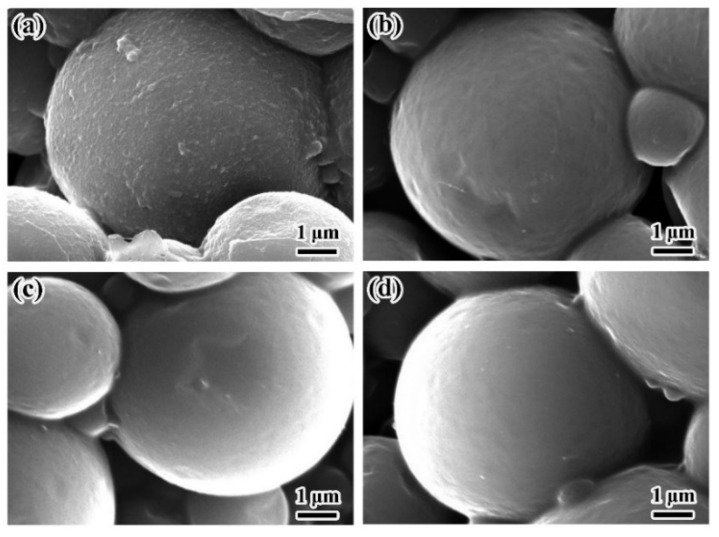
SEM image showing morphologies of microcapsules prepared at different emulsifier dosages: (**a**) 1.5%, (**b**) 2.0%, (**c**) 2.5%, and (**d**) 3.0%.

The data in Table 1 and Figure 3a show that when the emulsifier dosage was less than 1.5% of the whole system, microcapsules would adhere to each other during polymerization. When the dosage of emulsifier was higher than 1.5%, the obtained microcapsules were well dispersed. A smooth surface was obtained when the dosage was greater than 2.0% (see Figure 4b–d). The morphology of microcapsules is related to the degree of emulsification in the emulsion. Different amounts of emulsifier can lead to different surface tension behavior on the oil–water interface. More emulsifiers would lead to lower surface tension of the aqueous phase, smaller droplets, and more stable emulsions are easier to form after mixing, thereby avoiding or reducing the surface damage or wrinkle caused by oil phase dissolution during the formation of microcapsules. The D_50_ of the particles showed regular changes and decreased with increasing emulsifier dosage. At emulsifier dosages greater than 2.5%, there were no obvious changes in the D_50_. Figure 3 shows OM images of two samples prepared at different emulsifier dosages.

### 3.2. Effects of Heating Rate during Consolidation on Encapsulation

Because of the presence of active –NH_2_ groups in its structure, COS can be viewed as a polyamine. Isocyanates react with polyamines to give polyurea microcapsules via interfacial polymerization; the main reactions are as follows [43,44]:

(i) The –NCO group reacts with –NH_2_ to form polyurea.
(ii) Isocyanate is hydrolyzed in water to form carbamic acid, which is unstable and rapidly breaks down to CO_2_ and an amine.
(iii) The amine produced by isocyanate hydrolysis continues to react with isocyanate and forms polyurea.

**Figure 5:**
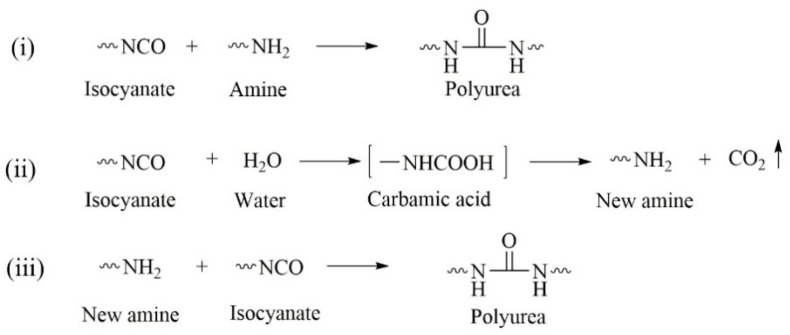
Main reactions in preparation of polyurea microcapsules by interfacial polymerization of isocyanate and amine.

The entire process is shown in Figure 5.

**Figure 6:**
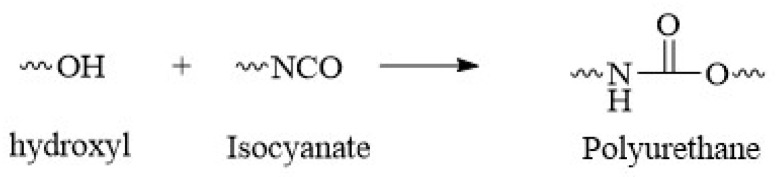
Formation of polyurethane from isocyanate and hydroxyl.

In addition to –NH_2_, there are –OH groups in the molecular structure of COS. The activity of –OH is much lower than that of –NH_2_. Nevertheless, along with polyurea formation, a small proportion of –NCO can react with –OH to form polyurethane. This was confirmed by IR spectroscopy. The reaction is shown in Figure 6.

During polymerization at the interface, the core material is encapsulated into the formed polymer shell. The heating rate of the reaction system during the consolidation process has an important effect on microencapsulation. In this study, the initial temperature was room temperature, and the system was heated at 65 °C for 6 h. The impact of the heating rate on microencapsulation was investigated.

**Figure 7:**
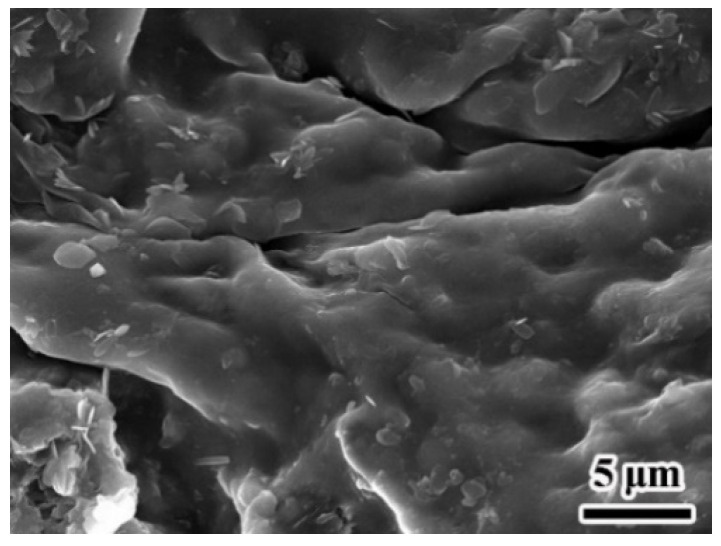
Agglomeration of MDI and COS.

As shown in Figure 5, isocyanate monomers can be hydrolyzed to an amine and CO_2_ at the emulsion interface. The CO_2_ escapes rapidly from the system because of its low solubility. The hydrolysis rate increases with increasing temperature [45]. If the reaction is too fast, agglomeration occurs and microcapsules cannot be obtained (Figure 7). More than that, too much CO_2_ might be formed in a short time, which could cause rapid release of material from the system and might even be dangerous. The experimental results shown in Table 2 indicate that the ideal heating rate is 3–5 °C/10 min.

### 3.3. Effects of Agitation Rate during Emulsion Preparation on Microcapsules

**Figure 8:**
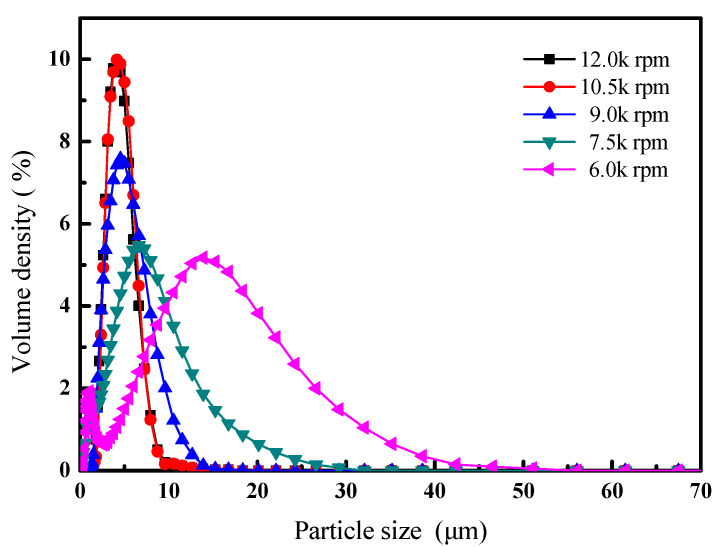
Relationship between agitation rate during emulsion preparation and size distribution of obtained microcapsules.

The agitation rate during emulsion preparation is one of the main factors that affect the microcapsule particle size distribution. A high agitation rate produces a high break-up force, which generates microemulsions with small, stable droplets. A high-speed shearing machine was used to obtain such an ideal emulsion. Figure 8 shows the relationship between the agitation rate during emulsion preparation and the microcapsule particle size distribution.

Figure 8 and Table 3 show that there is a relationship between the agitation rate and particle size distribution: as the agitation rate during emulsion preparation increased, the microcapsule D_50_ decreased from 10.72 to 4.20 μm, and the RS decreased from 2.27 to 0.93. This is because increasing the agitation rate causes formation of smaller and more stable O/W emulsions.

The specific surface area of a particle is closely related to the particle size, therefore smaller microcapsules have larger specific surface areas and higher release rates [41]. The microcapsule particle size and particle size distribution can therefore be changed by adjusting the agitation rate during emulsion preparation, and the release rate of the microcapsules can be regulated.

### 3.4. Effects of Core/Shell Ratio on Microencapsulation

The core/shell ratio affects properties such as encapsulation efficiency, loading capacity, and release behavior. In this section, the effects of the core/shell ratio on the encapsulation efficiency and loading capacity are discussed.

**Figure 9:**
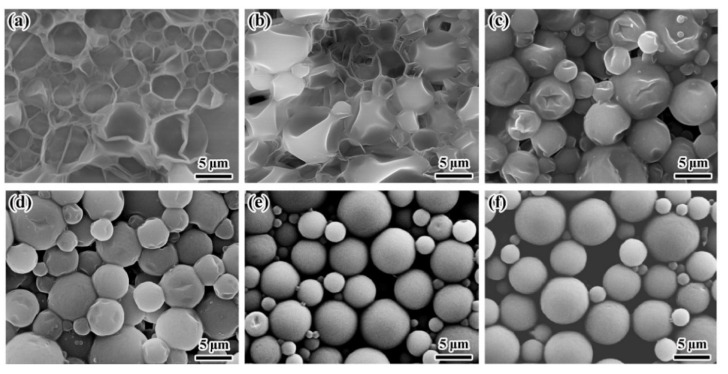
SEM micrographs of microcapsules with various core/shell mass ratios (m/m): (**a**) 8.0/1.0, (**b**) 8.0/2.0, (**c**) 8.0/3.0, (**d**) 8.0/4.0, (**e**) 8.0/5.0, and (**f**) 8.0/6.0.

Figure 9 shows that the prepared microcapsules gradually became spherical with increasing amounts of shell material. When the core/shell ratio was 8.0/3.0 or 8.0/4.0, the microcapsule surfaces were wrinkled and depressions were clearly visible (Figure 9c,d). When the core/shell ratio was 8.0/5.0 or 8.0/6.0, the obtained microcapsules were spherical and no collapsed microcapsules were observed (Figure 9e,f). This phenomenon was due to the amount of shell materials used. When the amount of shell was less, the shell of the microcapsule was too thin to have enough mechanical strength and compactness after drying, resulting in cracking or collapse. With more wall material used, more MDI and COS were involved in the reaction, microcapsule shells would become more compact, and the mechanical strength and compactness of the microcapsules would be improved.

**Figure 10:**
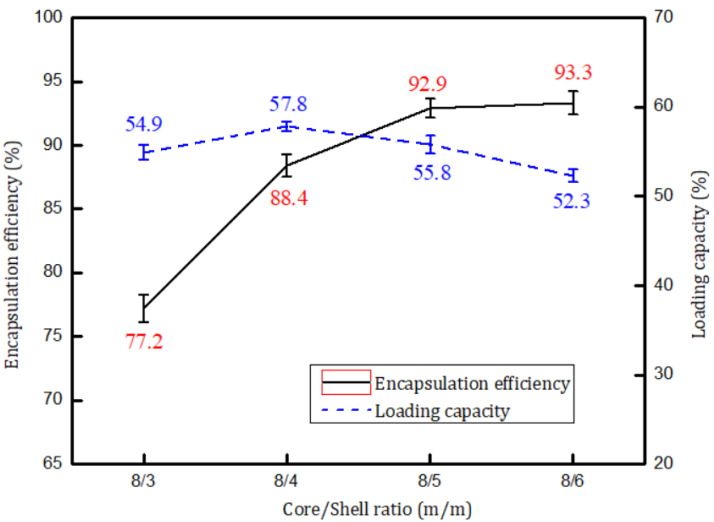
Encapsulation efficiencies and loading capacities at various core/shell mass ratios.

Figure 10 shows that the encapsulation efficiency gradually increased with increasing proportion of shell material. When the core/shell ratio was increased to 8.0/5.0, the encapsulation efficiency did not improve significantly. When the core/shell ratio was 8.0/6.0, 93.3% of the encapsulation efficiency, the highest value in this study, was obtained. However, the trend in the loading capacity was not consistent with the encapsulation efficiency. Initially, the loading capacity increased with increasing the amount of shell material. Compared with the core, the proportion of shell increased faster with the increase of the amount of shell materials, resulting in a downward trend of LC. The highest loading capacity, i.e., 57.8%, was achieved when the core/shell ratio was 8.0/4.0.

### 3.5. FT-IR Spectroscopy

**Figure 11:**
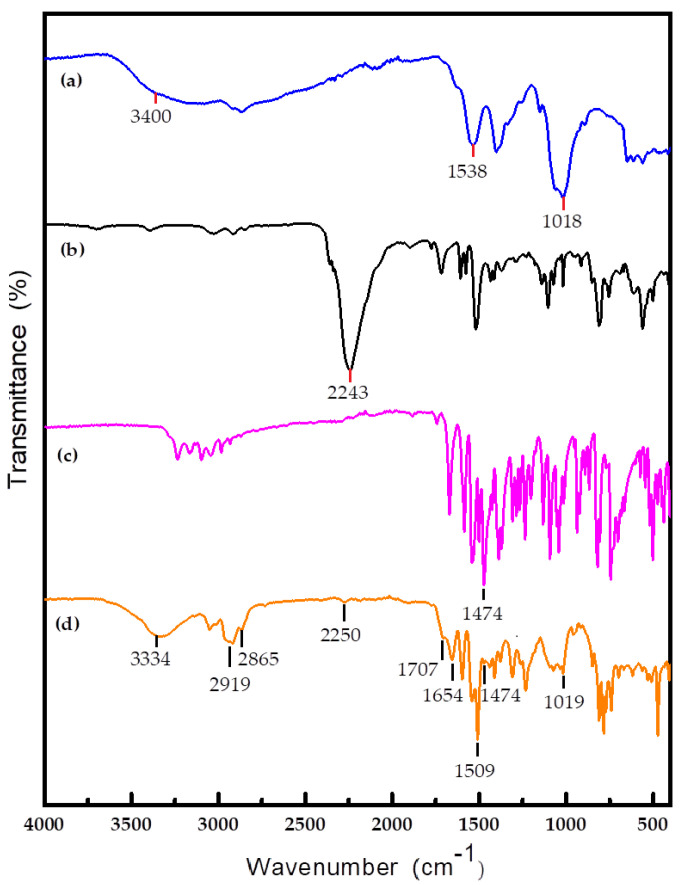
FT-IR spectra of COS (**a**), MDI (**b**), 2-((1-(4-chlorophenyl)-1*H*-pyrazol-3-yl)oxy)-*N*- (3,4-dichlorophenyl)propanamide (**c**), and microcapsule (**d**).

Fourier transform infrared (FT-IR) spectroscopy was used to investigate polymerization of the shell material and verify encapsulation of the core material. Figure 11 shows the IR spectra of the core material, COS, MDI, and microcapsules in the range 400–4000 cm^−1^.

Figure 11a shows the FT-IR spectrum of COS. The band at around 3400 cm^−1^ is assigned to N–H and O–H stretching, the peak at 1538 cm^−1^ represents in-plane bending of N–H. The peak at 2243 cm^−1^ in the MDI spectrum (Figure 11b) corresponds to the isocyanate group. The spectrum of the core material is shown in Figure 11c. Because of the conjugation effect, the pyrazole C=N framework vibrations give an absorption peak at 1474 cm^−1^. The spectrum of the obtained microcapsule is shown in Figure 11d. The absorption band at 2243cm^−1^ that is observed in the MDI spectrum almost disappears from the microcapsule spectrum. This indicates complete reaction of MDI. The absorption peak at 1654 cm^−1^ is assigned to the C=O stretching vibration of –NHCONH–. This indicates formation of polyurea via a reaction between the –NCO in MDI and –NH_2_. The peak that appears at 1707 cm^−1^ is assigned to C=O stretching of –NHCOO–. This shows that –NCO in MDI reacted with –OH in COS, and a small amount of polyurethane was formed. The peaks at 2919 and 2865 cm^−1^ correspond to the C–H stretching vibration of the aliphatic methylene group. The band near 1019 cm^−1^ is attributed to the aliphatic ether C–O–C stretching vibration (COS). This indicates the presence of COS in the shell material. The peaks at 3334 and 1035 cm^−1^ correspond to the O–H stretching and in-plane deformation vibrations of terminal O–H groups. This indicates that many –OH groups did not participate in the reaction. The peak at 1474 cm^−1^ in the core material spectrum (Figure 11c) is considerably weaker in the microcapsule spectrum. This shows that most of the core material was encapsulated in the microcapsules, but some of it was exposed on the microcapsule surfaces. The peak near 1509 cm^−1^ is assigned to the benzene skeleton vibration, which comes from MDI.

### 3.6. TGA

**Figure 12:**
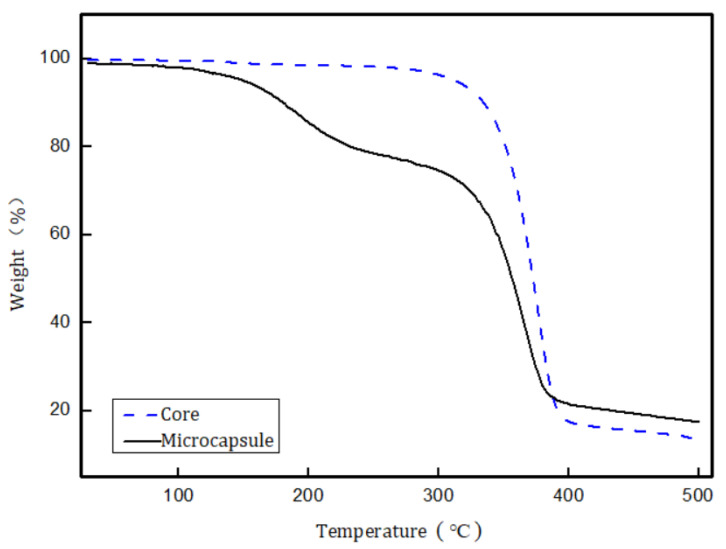
TGA curves for core material and obtained microcapsules.

TGA can be used to evaluate the thermal stability of a sample. The TGA curves for the core material and the obtained microcapsules are shown in Figure 12.

Figure 12 shows that the obtained microcapsules underwent two main mass loss phases in the investigated region, i.e., 25–500 °C. The first mass loss, at 140 to 308 °C, originates from decomposition of the shell material. The second mass loss, at 304–404 °C, is attributed to volatilization of the core material. This two-stage mass loss provides clear evidence of core material encapsulation. The onset temperature for shell material decomposition was much lower than that for core material volatilization. This indicates that the thermal stability of the shell material was lower than that of the core material. The shell material can therefore be degraded and the core material can be completely released. The use of such shell materials for pesticide microcapsules decreases pesticide residues, environment pollution, and hazards to humans.

### 3.7. Release Behavior of Microcapsules

**Figure 13:**
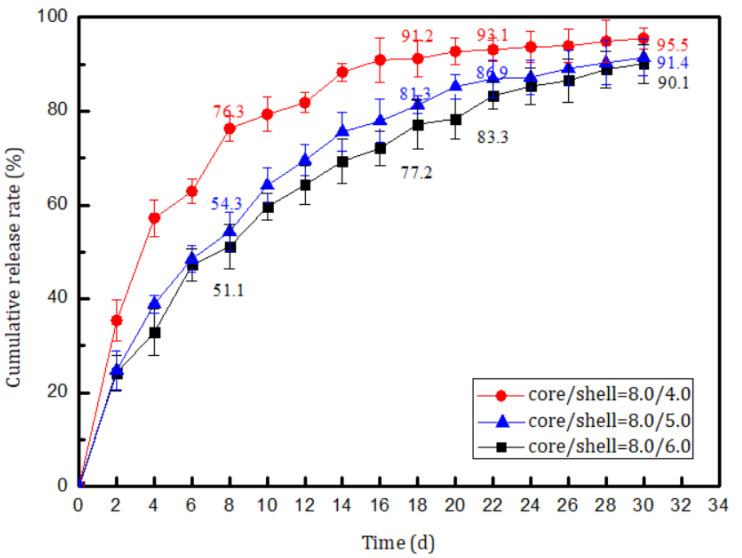
Release behaviors of microcapsules with different core/shell ratios.

The slow-release capabilities of three samples with different core/shell mass ratios (8.0/4.0, 8.0/5.0, and 8.0/6.0) were investigated. A plot of the cumulative release rate of core material vs. time is shown in Figure 13.

Figure 13 shows that the cumulative release rate of the core material increased with time. The release curves for all three samples showed a similar trend. Initially, the cumulative release rates for the three samples increased rapidly; they exceeded 50% on day 8 and 75% on day 18. This indicates that large amounts of core material were released from the microcapsules. The curves flattened after about 20 days. This is mainly because the amount of core material in the microcapsule was higher during the initial stage of the test. As the amount of core material in the capsule decreased, the curves flattened. This indicates that the release rate decreased and then became constant. The results clearly show that a higher core/shell ratio gave a higher accumulative release rate. On day 30, the accumulative release rates of the microcapsules with core/shell mass ratios of 8.0/4.0, 8.0/5.0, and 8.0/6.0, reached 95.5%, 91.4%, and 90.1%, respectively. Adjusting the core/shell ratio therefore enables the preparation of microcapsules with an ideal release rate.

### 3.8. Release Kinetics Model Fitting

The slow-release process of the microcapsules was further investigated by fitting the release data for microcapsules with different core/shell ratios to the core material release kinetic equation.

Zero-order kinetic equation:

Y = Y_0_ + k_0_t, (5)

First-order kinetic equation:

(6) $$Y=Y_{1}-(1-\frac{M}{Y_{1}}e^{\mathrm{kt}}),$$

Higuchi equation:

Y = KHt 1/2(7)

where t is the release time, Y is the percentage of core material released at t, Y_0_ is the initial concentration of core material in solvent (it is usually zero), Y_1_ is the maximum cumulative release percentage, and k_0_, m, k, and K_H_ are kinetic constants.

**Figure 14:**
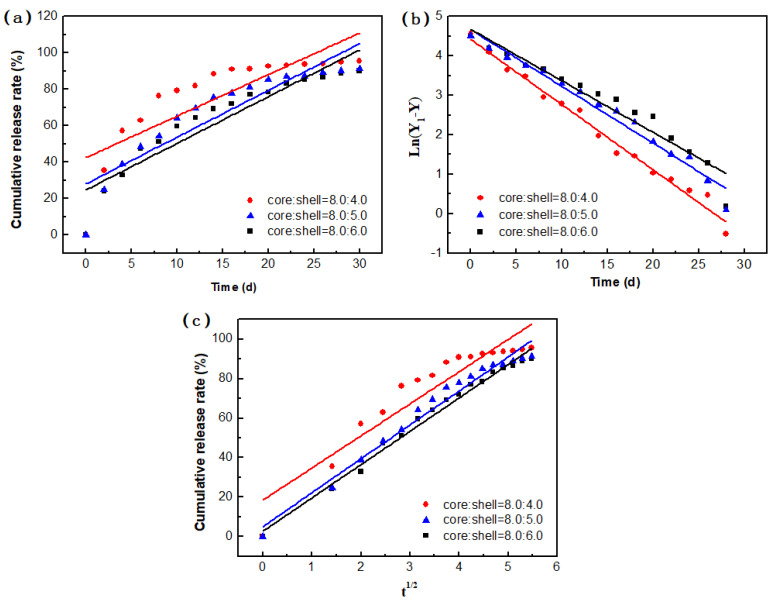
Kinetic models of microcapsule release: (**a**) zero-order kinetic model, (**b**) first-order kinetic model, and (**c**) Higuchi model.

The fitting results for the three kinetic models are shown in Figure 14 and Table 4.

As shown in Table 4, the fitting results for the first-order kinetic model followed well the linearity, with the R^2^ value higher than 0.937. The release process is ascribed to the core concentration. The core material inside can pass through the shell because of the concentration difference. In the initial stage (particularly in the first 7 days), there are more core materials in the microcapsule, and the concentration difference between the inside and outside of the microcapsule is larger, so the release rate is faster. With the decrease of core material, the release rate gradually slowed down with time. There was no sudden release during the whole test. Such a diffusion process shows that the structures and pore sizes of the microcapsules in this process were stable, and there was no sudden degradation or collapse.

## 4. Conclusions

In summary, microencapsulates of a new compound with fungicidal activity against *Rhizoctonia solani*, 2-((1-(4-chlorophenyl)-1*H*-pyrazol-3-yl)oxy)-*N*-(3,4-dichlorophenyl)-propanamide was prepared by interfacial polymerization of MDI and COS. Several parameters that affected encapsulation efficiency were optimized. FT-IR and TGA showed that the core material was successfully encapsulated in polyurea. When the core/shell ratio was 8.0/6.0, the encapsulation rate was 93.3%. In addition to a high encapsulation rate, microcapsules with a smaller particle size and narrower particle size distribution were obtained by adjusting the preparation parameters. Investigation of the release behavior showed that the microcapsules gave well-controlled release of the core material, and the release behavior could be fitted well with first-order kinetics. As COS has many high biological activities as well as biodegradability and a pure nature, microcapsules formed from COS are promising candidates for applications in controlling release of pesticides, growth regulators, and fertilizer.

## Figures and Tables

**Table 1:** Effects of emulsifier dosage on encapsulation.

No.	Dosage (%, m/m) ^1^	Material Status	Surface of Microcapsule	D_50_ (μm)
1	0.5	agglomeration	/	/
2	1.0	agglomeration	/	/
3	1.5	dispersed	rough	7.41 ± 0.95
4	2.0	dispersed	smooth	4.70 ± 0.71
5	2.5	dispersed	smooth	4.28 ± 0.43
6	3.0	dispersed	smooth	4.23 ± 0.29

^1^ Dosage as a proportion of the whole system. Conditions: The emulsion agitation rate was 10.5k r/min; the core/shell ratio was 8.0 to 5.0 (m/m).

**Table 2:** Effects of heating rate during consolidation on microencapsulation.

No.	Heating Rate °C/10 min	Reaction	State
1	20	vigorously	agglomeration
2	10	vigorously	agglomeration
3	5	mildly	dispersion
4	3	mildly	dispersion

**Table 3:** The D_50_ and RS for six microcapsule samples.

No.	Agitation Rate/rpm	D_50_ (μm)	RS
1	6.0 × 10^3^	10.72 ± 0.94	2.27
2	7.5 × 10^3^	6.08 ± 0.80	1.66
3	9.0 × 10^3^	4.77 ± 0.65	1.20
4	10.5 × 10^3^	4.28 ± 0.43	0.95
5	12.0 × 10^3^	4.20 ± 0.32	0.93

**Table 4:** Fitting results for microcapsule release.

No.	Core to Shell Ratio (m/m)	Goodness of Fit (R^2^)		
		Zero-Order Elimination Kinetics	First-Order Kinetic	Higuchi
1	8.0:4.0	0.648	0.986	0.879
2	8.0:5.0	0.824	0.973	0.971
3	8.0:6.0	0.866	0.937	0.986

## Data Availability

Not applicable.

## Supplementary Materials

The following are available online at https://www.mdpi.com/article/10.3390/ma14133753/s1, Figure S1: The curve of core material absorbance versus wavelength, Figure S2: Absorbance versus concentration calibration curve for core material at 284 nm.
