# Supplementary material for: Preparation of Polyurea Microcapsules by Interfacial Polymerization of Isocyanate and Chitosan Oligosaccharide
Source: Materials (Basel). 2021 Jul 5;14(13):3753. doi: 10.3390/ma14133753 (PMC8269919; doi:10.3390/ma14133753)
Supplement: Supplementary file 1 [file materials-14-03753-s001.zip › materials-1239804-supplementary.pdf]

materials
MDPI

Supplementary Materials

Preparation of Polyurea Microcapsules by Interfacial Polymerization of Isocyanate and Chitosan Oligosaccharide

Fuqiang Yu [1,2], Ying Wang [2], Yan Zhao [3,*], Jingyu Chou [2] and Xiaowu Li [1,*]

[1] Department of Materials Physics and Chemistry, Key Laboratory for Anisotropy and Texture of Materials, School of Material Science and Engineering, Ministry of Education, Northeastern University, Shenyang 110819, China; yufuqiang2003@163.com
[2] State Key Laboratory for the Discovery and Development of Novel Pesticides, Shenyang Sinochem Agrochemicals R&D Co. Ltd., Shenyang 110021, China; wangying09@sinochem.com (Y.W.); choujingyu@sinochem.com (J.C.)
[3] Jihua Laboratory, Nanhai, Foshan 528200, China
* Correspondence: zhaoyan@jihualab.com (Y.Z.); xwli@mail.neu.edu.cn (X.L.)

Construction of absorbance–concentration standard curve

Figure S1 shows that in the range 190–700 nm, a toluene solution of the core material showed an absorbance at 284 nm. The detection wavelength for the core material was therefore taken to be 284 nm.

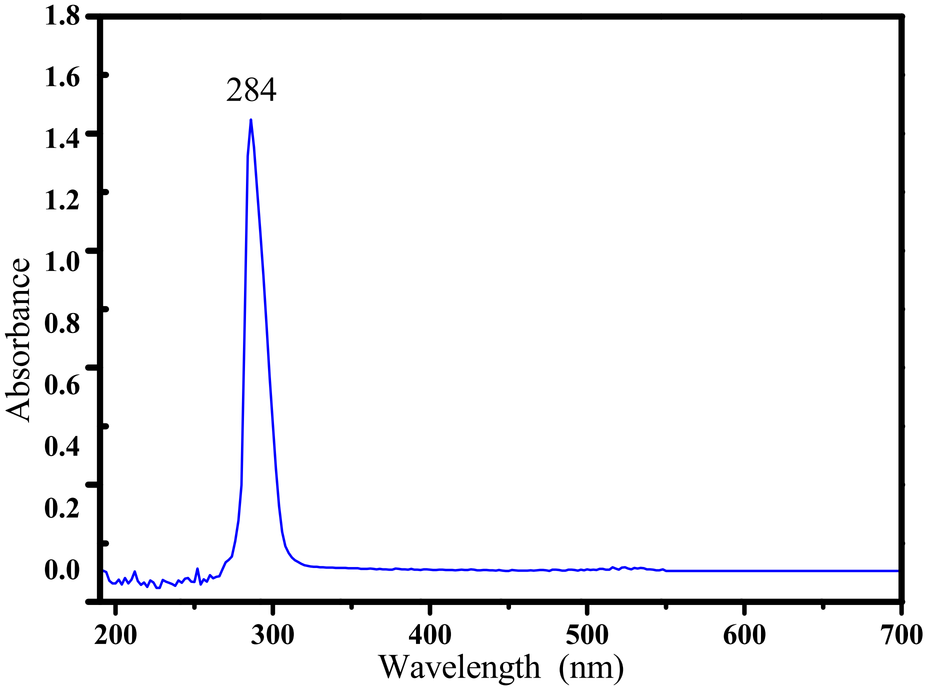

Figure S1. Core material absorbance versus wavelength plot.

The pure core material (100 mg, *d* = 0.0001 g) was dissolved in toluene (100 mL). The solution was diluted with toluene to 1.6, 3.2, 6.4, 9.6, 12.8, 16.0, and 19.2 mg/L. The absorbance of each sample was determined at 284 nm and the data were used to construct a standard curve. The absorbance at each concentration was determined three times and averaged.

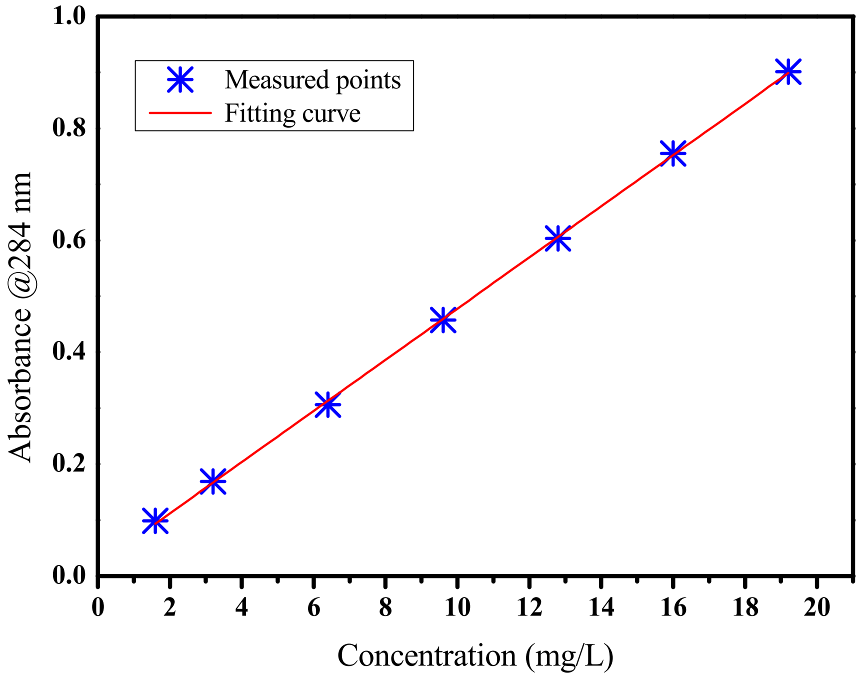

Figure S2. Absorbance versus concentration calibration curve for core material at 284 nm.

Figure S2 shows that the mass concentration of the core material was linearly proportional to its absorbance between 1.6 and 19.2 mg/L. The relationship between the absorbance intensity (Abs) and mass concentration of the core material (C) is Abs = 0.04575C + 0.02038; for the standard curve, the correlation coefficient R^2 = 0.99977.
